# Effect of Different Wavelengths of Laser Irradiation on the Skin Cells

**DOI:** 10.3390/ijms22052437

**Published:** 2021-02-28

**Authors:** Aleksandra Cios, Martyna Ciepielak, Łukasz Szymański, Aneta Lewicka, Szczepan Cierniak, Wanda Stankiewicz, Mariola Mendrycka, Sławomir Lewicki

**Affiliations:** 1 Department of Microwave Safety, Military Institute of Hygiene and Epidemiology, 04-141 Warsaw, Poland; aleksandracios@gmail.com (A.C.); ciepielakmartyna@gmail.com (M.C.); wanda.stankiewicz@gmail.com (W.S.); 2 Department of Molecular Biology, Institute of Genetics and Animal Biotechnology, Polish Academy of Science, Postępu 36A, 05-552 Magdalenka, Poland; 3 Laboratory of Food and Nutrition Hygiene, Military Institute of Hygiene and Epidemiology, Kozielska 4, 01-163 Warsaw, Poland; aneta.lewicka@wihe.pl; 4 Department of Patomorphology, Military Institute of Medicine, Szaserów 128, 04-141 Warsaw, Poland; scierniak@wim.mil.pl; 5 Faculty of Medical Sciences and Health Sciences, Kazimierz Pulaski University of Technology and Humanities, 26-600 Radom, Poland; mariolamendrycka@poczta.onet.pl; 6 Department of Regenerative Medicine and Cell Biology, Military Institute of Hygiene and Epidemiology, 04-141 Warsaw, Poland; lewickis@gmail.com

**Keywords:** laser irradiation, skin exposition, dermal effect of laser, UV, IR, laser treatment

## Abstract

The invention of systems enabling the emission of waves of a certain length and intensity has revolutionized many areas of life, including medicine. Currently, the use of devices emitting laser light is not only an indispensable but also a necessary element of many diagnostic procedures. It also contributed to the development of new techniques for the treatment of diseases that are difficult to heal. The use of lasers in industry and medicine may be associated with a higher incidence of excessive radiation exposure, which can lead to injury to the body. The most exposed to laser irradiation is the skin tissue. The low dose laser irradiation is currently used for the treatment of various skin diseases. Therefore appropriate knowledge of the effects of lasers irradiation on the dermal cells’ metabolism is necessary. Here we present current knowledge on the clinical and molecular effects of irradiation of different wavelengths of light (ultraviolet (UV), blue, green, red, and infrared (IR) on the dermal cells.

## 1. Introduction

Lasers (**L**ight **A**mplification by **S**timulated **E**mission of **R**adiation) are widely used tools in science, medicine, and industry. The most popular application of lasers is as a cutting tool. The first study was reported in 1967 by P. Houldcroft, who used a CO_2_ laser with oxygen to cut a 1 mm thick steel sheet [1]. Currently, lasers are used worldwide to precisely cut all sorts of shapes, including 3D forms, in various materials [2]. The cutting properties of lasers are also used in medicine surgery, where precision is the most important [3]. The second application of lasers is the area of diagnostics. The lasers enabled the creation and development of devices which by using the fluorescence phenomena combined with the monoclonal antibodies methodology became a powerful tool to study cell metabolism and functions [4]. The third popular application of lasers is the treatment of various diseases (medicine) or correction of the inaccuracies of the body (cosmetology). Low power lasers which affect cellular metabolism are usually used in this application [5,6,7]. The clinical trial website (https://clinicaltrials.gov/ (accessed on 1 February 2021)) lists over 2850 studies, which use or used lasers for the treatment of different diseases. 

The emitted laser light has the form of a much focused, parallel monochromatic beam (with one specific wavelength) with very high intensity. The laser light differs from the light emitted by other sources by the beam, which is consistent, coherent, and its rays have the same wavelength [8]. Lasers irradiation is described by several parameters. The wavelength is the most important. It determines the depth of the penetration by the light—the higher the wavelength, the greater the laser penetration through the tissues [9]. This is also associated with the thermal effect caused by the light which increases with the increasing wavelength. The next parameters are the density of the laser energy, and the duration of radiation. Both parameters specify the general laser irradiation dose absorbed by the cells, which in turn differently affect the cell metabolism [10]. The last parameter of the laser impact on biological tissues is a type of impulse: continuous or pulsating. 

Because there is a lot of information on the laser effect on the human and animal tissues, here we focus on the skin cells. We describe the mechanisms of action of ultraviolet (UV), blue, green, red, and infrared (IR) lasers on the cells cultured in vitro, and the treatment of skin diseases. 

## 2. Lasers and LEDs

Currently, both lasers and LEDs are used in medicine. The major difference between these light sources is that the lasers have narrow spectral width (around 1 nm) which means that the light is emitted in form of a single wavelength while LEDs have a spectral width of up to 80 nm which results in a more broad, Gaussian-like spectrum of emitted light. What is more, LEDs have higher beam divergence than lasers. Due to the characteristic of emitted light, the laser operating at the same nominal power and wavelength that the corresponding LED will deliver more energy to the target. Therefore, scientific results obtained using LED in an experimental setting cannot be treated equally to the result obtained with corresponding (power and wavelength) laser. Nevertheless, LEDs and lasers utilizing the same wavelength promote similar biological effects but with varying effectiveness [11]. In the study of NIR-PIT (near-infrared photoimmunotherapy), Sato and et al. showed that laser light had superior cytotoxic efficacy to LED light at the same energy levels in both 2D and 3D-spheroid cell cultures in vitro. What is more, laser light produced better therapeutic effects than LED light in NIR-PIT in vivo at the same light dose in mouse models [12]. Another limitation of LEDs is that some NIR-PIT applications, especially in oncology, require light to be delivered through catheters, endoscopes, or needles in which case a coherent narrow beam laser light is preferred. 

## 3. Laser Interaction with a Skin

**Figure 1:**
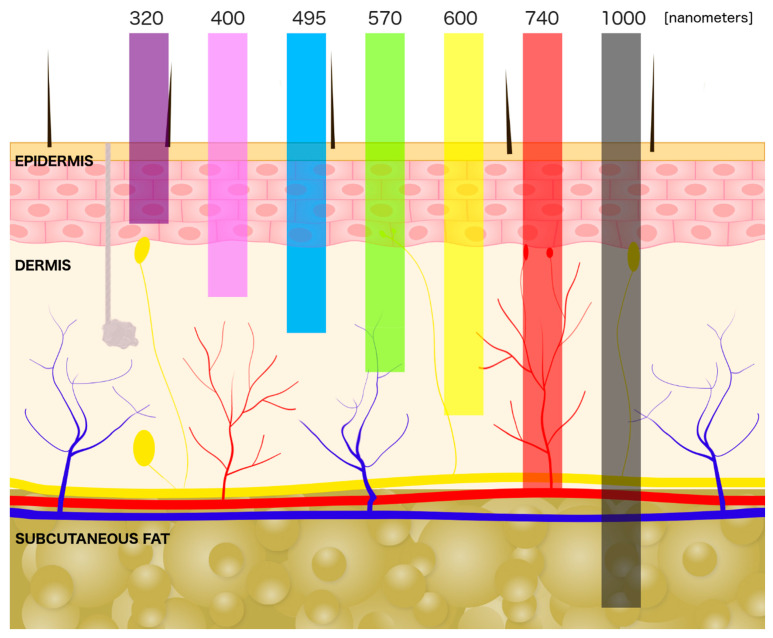
The skin cross-section showing dermal penetration by different wavelengths of light (in order from the left: UVB, UVA, blue light, green light, yellow light, red light, infrared light).

Human skin exhibits specific properties that determine the penetration and absorption of laser light by skin cells. The light generated from laser devices interacts with the tissue in four different ways: transmission, reflection, scattering, and absorption [13]. The most important for the biological effect of laser light is absorption. The tissue absorbs photon energy which, in turn, as radiant energy can be reemitted or transformed into heat, and increase the internal temperature of the tissue [14,15,16]. In the skin, the absorption of laser light is dependent on the interaction with the different chromophores—endogenous compounds which absorb specific wavelengths. Water, melanin, and hemoglobin are three primary endogenous cutaneous chromophores [8,17]. Moreover, laser scattering in the biological tissue determines the intensity of light energy [18]. The scattering amount of energy of the laser is inversely proportional to the wavelength. The penetration of laser light in biological tissue increases with wavelength up to mid-infrared, where water that present in the tissues, absorbs the most energy of laser light. [17] (Figure 1). 

**Figure 2:**
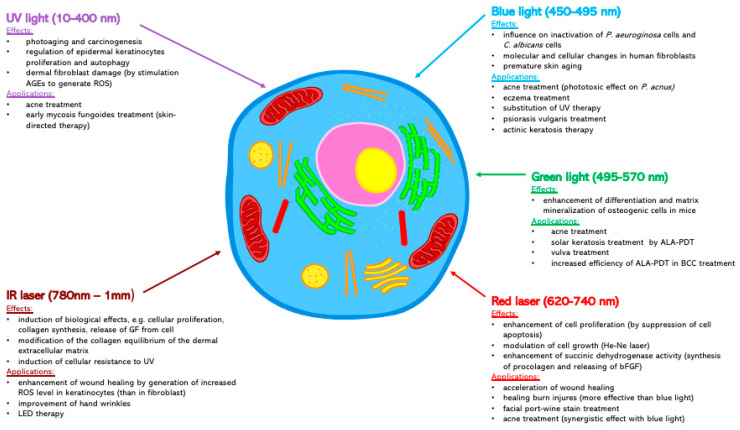
Summary of the most important properties and effects on the cell of various wavelengths of light. AGEs—advanced glycation end-products; ALA-PDT—aminolevulinic acid photodynamic therapy; BCC—basal cell carcinoma; bFGF—basic fibroblast growth factor; GF—growth factor; HMME-PDT—hematoporphyrin monomethyl ether photodynamic therapy, LED—light-emitting diode, ROS—reactive oxygen species.

More specific knowledge of this interaction between laser and skin can help the specialist to select specific laser parameters in their therapies, such as the wavelength of the laser light, intensity, duration of radiation, and the density of laser beam energy [19]. Some medical uses of lasers are presented in Figure 2. 

## 4. The UV Light (10–400 nm)

The UV radiation, the universal source of non-ionizing radiation, emitted by the sun, is essential for life and its development on Earth. The UV radiation is divided into three ranges with different biological properties: UVA (320–400 nm), UVB (280–320 nm), and UVC (200–280 nm). The ozone layer of Earth blocks the majority of UVC but only 5% of UVB radiation. Part of the UVB radiation not blocked by the ozone layer penetrates only the surface layers of the epidermis and can reach the upper papillary dermis. Nevertheless, the harmful effects of UVB should not be neglected [20,21]. In contrast, the UVA radiation is absorbed by the deeper layers of the dermis. 

There are three main types of UV lasers. First, the Q-switched Nd:YAG laser in which ultraviolet 353 nm laser irradiation is changed from 1064 nm (infrared) wavelength using a special crystal. The second type is a gas (excimer) laser, commonly used in the treatment of psoriasis [22,23]. The third type is a metal vapor laser [24]. 

UV radiation is known to be responsible for adverse effects on the skin such as cell damage, photoaging, and carcinogenesis [25,26]. Additionally, the absorption of the radiation by skin chromophores or the formation of reactive free radicals (reactive oxygen species - ROS) from the water present in the skin causes premature skin aging, modified pigmentation, and a loss of collagen [27,28,29]. UV radiation affects mainly keratinocytes, which in turn begin to release pro-inflammatory cytokines: IL-1α, IL-1β, and IL-6. Specific, interrelated secretion cycles of the proinflammatory cytokines induce synthesis and release of matrix metalloproteinase 1 (MMP-1). Due to its inflammatory action, UVB, with its long-lasting effect, destroys the internal microstructure of the skin, resulting in its faster aging [30]. Interestingly, Gruber et al. [31] showed similar skin effects of UVB radiation in the in vitro reconstructed human skin models such as MatTek EpiDerm. The cells after irradiation increased secretion of pro-inflammatory cytokines (IL-1α, IL-6, IL-8), and prostaglandin in comparison to the control group. These results were confirmed by Penna et al. [32]. Taken together, these confirm the hypothesis that in some cases reconstructed skin tissues may be a good substitute for conducting effective skin tests, especially when the morphology of natural skin is preserved, [33]. It has been shown that exposure to UV light might regulate epidermal keratinocytes autophagy through selective down-regulation of key autophagy-related genes including ULK1, ATG3, ATG5, and ATG7 [34]. In fibroblasts, UVB irradiation proved to cause a significantly higher production of ROS, DNA damage, and mitochondrial impairment resulting in activation of many pathways leading to apoptosis, decreased proliferation of cells, and skin fibrosis [32,35]. 

Micka-Michalak et al. [21] proposed that UVB light (308 nm, 250 mJ/cm^2^) during early post-irradiation induces temporary immune and angiogenic responses in pigmented skin analog prepared from cell lines isolated from patients. The examination of the local cellular response showed a moderate cell proliferation in the dermis of skin analog [21]. Kwon et al. [36] showed that the LED light, emitting UVA and UVB light (310 and 340 nm), used on NC/Nga mice with atopic dermatitis significantly alleviated the disease-related lesions (itching, dryness, erythema, and edema). UV-LED phototherapy attenuates the secretion of the proteins responsible for atopic dermatitis (AD) such as IL-1a, IL-1β, IL-31, ICAM-1 protein, and E-selectin, which reduced the infiltration of the mast and inflammatory cells while weakening acanthosis and keratosis in the studied mice. UVA, because it does not penetrate deep into the skin layers, can affect AD cells associated with pathogenesis, thereby causing T cell apoptosis and reducing the number of mast and Langerhans cells. While UVB depletes Langerhans cells it regulates the immunological activity of the skin in AD. Researchers suggested that narrow-spectrum UV-LEDs could be a good therapeutic tool in AD [36]. A summary of the effects of UV laser on skin cells is presented in Table 1.

## 5. Blue Light (450–495 nm)

The blue light contributes to premature skin aging by inducing molecular and cellular changes in human fibroblasts [37]. Fibroblasts shrink and become round, they have a disorganized cytoskeletal network and do not spread to the same extent as non-irradiated cells. Moreover, the expression of genes related to mitochondria and actin cytoskeleton decreases. Nakashima et al. [38] support the theory that direct and prolonged exposure to blue light, similar to UV light, contributes to skin aging and carcinogenesis. Research conducted on the hairless mice that express redox-sensitive GFP, exposed to blue light, showed a significant increase in oxidative stress. Similar results were obtained in HaCaT cell culture. It is possible, however, that the produced ROS, through flavin as a photosensitizer, is in the peroxide form. However, some researchers claimed that blue light has a positive effect on fibroblasts and may be used in keloids and fibrosis therapies [39,40]. The blue-light lasers are mostly used for acne treatment.

Zhang et al. [41,42] studied the effect of blue light (415 nm) on *Candida albicans* and *Acinetobacter baumannii* fungus in the keratinocytes culture and animal model. Their study suggests that there is a therapeutic window where the studied fungus is selectively inactivated by the blue light while the host cells (keratinocytes) remain unaffected. Wang. et al. [43] obtained similar results and concluded that 460 nm blue light wavelength eradicates the *C. albicans* biofilm in vitro. Additionally, while examining the effects of blue light (450 nm, 84 J/cm^2^) on methicillin-resistant Staphylococcus aureus, Makdoumi et al. [44] discovered that blue light was able to eliminate 70% of bacteria, without affecting immortalized human keratinocytes. Despite these promising results suggesting that antifungal properties of blue laser Mamalis et al. [45] showed that blue light-emitting diodes boosted, in a dose-dependent manner, the reactive oxygen species production, and inhibited proliferation and decreased migration speed of human fibroblasts.

Teuschl et al. [46] used 470 nm irradiation in their research on “injured” fibroblasts and keratinocytes. The proliferation rate of both tested cell lines decreased and the apoptosis and necrosis rate of fibroblasts increased. In contrast, de Alncar Frenandes Neto et al. [47] tested the effect of the blue light produced by LEDs on third-degree skin burns in 40 Wistar rats. To their surprise, these rats consumed more food than animals from the control group. Additionally, the angiogenesis index increased after 7 days of treatment, and the skin of treated animals began to re-epithalize, which might be correlated with higher food consumption. After about 2 weeks of burn wounds healing, the fibroblasts become the most numerous population of cells in the granulation tissue. Subsequently, the fibroblasts transformed into myofibroblasts, which have a high expression of α-smooth muscle actin (α-SMA). Interesting results were observed in the studies carried out by AlGhamdi et al. [48,49] on melanocyte cell cultures. In the first study, the blue light laser was found to be the most effective, and it greatly intensified melanocyte viability, proliferation, and migration. In the second, the differentiation of melanosome into melanocytes showed that the red light laser was more effective than the blue light [49]. The discrepancy of the obtained results can be explained by the difference in the parameters that were used in those studies. The first studies [48] used lower doses of blue light energy than the energy doses of the red light used in the second research [49].

Castellano-Pellicena et al. [50] evaluated the ability of blue light to activate opsins in order to heal skin wounds and restore human epidermal barrier function. To their surprise, blue light stimulated keratinocyte differentiation even though it did not cause their migration in a scratch wound assay. Data also suggested that the opsins 3 receptor acts as a receptor of a blue-light and might be an important factor to restore the skin barrier function.

In recent years, the use of lasers has been gaining application in various dermatological therapies including daylight-mediated photodynamic therapy (d-PDT). This technique is common in the treatment of actinic keratosis (AK) because it significantly reduces pain. Furthermore, d-PDT therapy allows the treatment of a large area of the skin, treating several lesions in the same patient and several patients at the same time. d-PDT has also been approved in the US and European countries as a therapy for Bowen’s disease, superficial basal cell carcinoma (sBCC), and in some cases thin nodular BCC. D-PDT is also considered a candidate for the treatment of acne vulgaris. Recently, researchers are trying to improve this technique and replace daylight with artificial sources. In a study by Marra et al. [51] scientists were using different light sources, including an artificial blue source, on the nude mice. Unfortunately, it has been shown that blue light has worse therapeutic effects. Blue light, based on Stat3 analysis and histopathological examination, turned out to be a poor PDT mediator. Thus, the authors hypothesize that blue light does not work equally to the PpIX absorption spectrum. It may be related to the fact that blue light does not penetrate deeply into the skin or is related to the 5-ALA diffusion rate. To achieve the most satisfactory effects of blue light treatment, one should think about the exact determination of the definite ratio of the depth of light penetration to the time of its use, and the ultimate shortening of the use. A summary of the effects of blue laser on skin cells is presented in Table 2.

## 6. Green Light (495–570 nm)

For skin treatment, green lasers are usually used in combinations with red and yellow light, especially in patients with acne. The green laser has been more widely used in clinical trials [53,54,55,56]. JalalKamali et al. [57] showed the therapeutic effect of 532 nm green light on basal cell carcinoma. There was a 30% decrease in the cell viability with the use of polarized light when compared to the non-polarized light. Scientists, however, do not specify what could be the exact reason for these differences highlighting the importance of accurate biochemical and even physical research on the molecular, interactional symmetries and most importantly arrangements of organelles.

Green light compared to blue and red light radiation enhanced IL-8, leptin, and VEGFC production. 518 nm irradiation also promotes the migration of HaCaT cells [58]. Similar results were shown in a different experiment where green light promoted a higher proliferation rate than red and infrared light [59] possibly by inducing-EGF and VEGF production [58,60]. 

Other studies conducted on 20 patients with head AK showed that 3 sessions of photodynamic therapy with red or green light already caused disease remission. However, patients who were treated with red light experienced more pain in the irradiated areas than patients treated with a green light. However, there is no literature comparing the effects of green and red light in dermatological practice. The authors of the article speculate that these differences in adverse effects such as pain or tingling, burning and paresthesia may result from a decrease in the number of abnormal cells in AK foci and, consequently, a decrease in accumulated PpIX. Green light does not penetrate as deeply into the skin as red light. It penetrates only the epidermis without irritating the nerve fibers. Interestingly, 50% of patients treated with red light had a recurrence of AK lesions in comparison to the patients treated with a green light [61].

All these studies indicate that the green light irradiation is safe and provides more promising results than the red or IR light irradiation on skin cell lines. A summary of the effects of green laser on skin cells is presented in Table 3.

## 7. Red Laser (620–740 nm)

Theodore Maiman constructed the first red light laser (ruby laser) in 1960 [62] which is still used for removing tattoos, birthmarks, and hair. 

Researchers underline that the laser’s photobiomodulation parameter should never exceed the standard recommendation of the American National Standards Institute while the thickness and color of the skin should be taken into consideration for choosing energy doses to provide the therapeutic effectiveness of red light laser [63]. Recent reports showed that the combination of red light and toluidine blue O has an inhibitory effect on biofilm formation and inhibits bacterial adhesion. Unfortunately, this combined therapy was toxic for fibroblasts and reduced cell spreading [64]. The commonly-used low-intensity red light source is the He–Ne laser, which emits red light at a wavelength of 632.8 nm. Heiskanen and Hamblin [65] support the idea that there is no difference in the biologic response to irradiation with coherent red laser light and noncoherent red light. Sperandino et al. [60] are convinced that this type of radiation has a positive effect on the proliferation of keratinocytes. 660 nm laser irradiation in energy dose from 3, 6 to 12 J/cm^2^ promoted HaCaT proliferation rate and increased expression of Cyclin D1. In Evans et al. [66] study red light had different effects on keratinocytes cultured with the addition of H_2_O_2_. On the one hand, the irradiated keratinocytes went back to viable actively proliferating cells, on the other, the red light promoted their survival possibly by reducing the amount of ROS and improving their proliferation rate.

Quite an unusual study on the effects of the red and blue laser was carried by Niu et al. [67] In this study, the effects of both 405 nm and 630 nm irradiations were tested on keratinocytes treated with curcumin. The authors state that this uncommon combination may be efficient in the regulation of proliferation and apoptosis rates of the treated cells. Leong et al. [68] support the hypothesis that red light is more effective than blue light. In 3D skin models, red wavelength induced the release of IL-4 which was not mediated by opsins or photooxidative mechanisms. Hyun-Soo et al. [69] in their article state that red light has protective effects on the skin against UVB radiation. They showed that red light modulates normal human dermal fibroblasts to increase the expression of genes responsible for enhancing the adaptive response to redox, inflammatory balance, and, additionally, those genes that play a major part in DNA repair processes. Song et al. [70] showed that the effects of red light irradiation on human fibroblast cells depend on many factors such as energy dose, the wavelength of used light, and cell culture conditions. Wavelengths from a spectrum of 630 to 660 nm are suspected to have the most advantageous effects on fibroblasts. 636 nm laser irradiation generated a much lower amount of ROS when compared to the nonirradiated cells [71]. Ayuk et al. [72] suggested that the more stressed the fibroblasts cells are the better they respond to photobiomodulation of 660 nm wavelengths. Not only the studied wavelength increased cell proliferation and viability but also helped in wound healing via accelerated migration rate [73]. Red laser irradiation enhanced the synthesis of procollagen, the expression of collagen, and the release of basic fibroblast growth factor. A summary of the effects of red laser on skin cells is presented in Table 4.

## 8. IR Laser (780 nm–1 mm) 

The most popular lasers used in skin treatments are those which emit infrared (IR) light. The spectrum of applications of IR laser is wide, especially in medicine. Commonly used devices emitting IR light are Er: Yag and Nd: Yag lasers. 

As with all lasers, the biological effects of light irradiation depend on the photoacceptor molecule. The two main types of chromophores for IR light are intracellular water and cytochrome c oxidase [75]. As the water electromagnetic absorption spectrum is mostly in the IR region the photon absorption of these spectra results in an increase in intracellular temperature [76]. Therefore, cell or tissue biological response to IR radiation is in part caused by the generated thermal effect.

In Goerge et al. [71] studies near IR (NIR) laser caused increased production of ROS in the primary dermal fibroblasts. NIR is also suggested as a plausible useful tool for future synergistic cancer phototherapy [77]. In contrast, Solmaz et al. [78] showed that the 809 nm wavelength had no positive effects on L929 fibroblasts when compared to the 635 nm laser irradiation. Keratinocytes seem to be more sensitive to IR laser irradiation than fibroblasts. After exposure to GaAlAs diode laser, keratinocytes produced more ROS than fibroblasts, which inversely correlated with an expression of catalase [79]. Erebium: Yag laser used in Schmitt et al. [80] research caused an increase of mRNA expression of several matrix metalloproteinases (MMP) and their inhibitors, chemokines, and cytokines in 3D skin models.

De Filippis et al. [81] used Nd:YAG laser on human dermal fibroblast (HDF) and human normal epidermal keratinocytes (HaCaT) cell lines. Researchers believe that Q-switched Nd: YAG laser can be used to fight photoaging. 1064 nm irradiation significantly increased filaggrin and transglutaminase expression in the HaCaT cell line. Moreover, irradiation used in this research induced the expression of pro-inflammatory cytokines genes (interleukins IL-6, IL-1α, and TNF-α) in keratinocytes. In HDF stimulated with irradiated keratinocyte-conditioned media the expression of MMP1 was downregulated, and, on the other hand, TGF-β increased after 24 h of UV radiation. In addition, the collagen, I, procollagen, and elastin expressions were upregulated compared to control.

The effect of photobiomodulation (PBM) on wound healing and microbial flora was examined in 20 male Wistar type II diabetic rats. On day 7 after wound formation, PBM (890 nm) used alone significantly reduced colony-forming units (CFU), improved wound healing speed, and joint movement in the affected limb. Asghari et al. [82] considered that PBM supports wound healing by the fact that immune cells, mainly neutrophils, and macrophages, reduce local oxygen consumption by stimulating leukocytes to increase their phagocytic activity and keratinocytes for differentiation.

A new, safe protocol for acne treatment using a laser was introduced by Bitter [83]. The new treatment protocol involves 6-8 sessions, each of which consists of three stages using only one device but different wavelengths in each stage. During the first stage high-power, blue light with a wide area of action was used to kill acne-causing bacteria. In the second, simultaneously yellow and red light, with a smaller area of action, were used to stimulate neocollagenesis and exert anti-inflammatory effects. During the last stage, Bitter used IR light to maintain the effects of treatment and prevent relapses. This protocol caused 80% of treated patients to clear completely or achieve at least a 75% improvement in their inflammatory acne. The first visible improvements appeared after 2 to 3 days of therapy sessions and additionally 1–2-year-old scars faded after 1 to 3 weeks post-treatment.

Even though IR laser irradiation effects are still being studied in vitro and in vivo on animal models and clinical cases, IR lasers found an application in the improvement of hand wrinkles skin tightness [84,85]. A summary of the effects of red laser on skin cells is presented in Table 5.

## 9. Conclusions

Lasers have a wide range of applications in medicine, especially in dermatology where stimulation of healing, reduction of apoptosis and necrosis, and skin rejuvenation are required. There are still debates on whether which laser lights wavelengths and/or their combination brings the greatest and the best results. Before conducting planned experiments, it is necessary to establish the proper parameters of laser devices to provide appropriate safety precautions. 

## Figures and Tables

**Table 1:** UV light (10–400 nm) effect on skin cells.

Author	Parameters	Material	Main Findings after Laser Treatment
Micka-Michalak et al. [21]	308 nm; 250 mJ/cm^2^	Pigmented human skin analog (fibroblasts, melanocytes, and keratinocytes isolated from healthy patients) engrafted to the skin of immuno-deficient female nu/nu rats	increased number of CD31+ blood vessels, increased number of granulocytes and monocytes/macrophages, there was no difference in the expression of TNFα, no changes in lymphatic microvasculature.
Tang et al. [22]	308 nm; 0.05, 0.075, 0.10, 0.125, 0.150 and 0.175 J/cm^2^	36 patients with psoriasis, self-control study	effective treatment of laser observed in the head, folds, back, abdomen and limbs (6 weeks observation), PASI score lowered after laser treatment (6 weeks observation).
Jobe et al. [26]	365 nm; 10, 50 and 100 mJ/cm^2^	Co-culture of dermal cells (keratinocytes and fibroblast) isolated from human skin (healthy and with melanoma)	DNA damage in keratinocytes after 10 and 100 mJ/cm^2^ of UVB, 100 mJ/cm^2^ of UVB irradiation was lethal for the majority of keratinocytes.
Goldstein et al. [29]	311–313 nm, starting dose - 0.2 J/cm^2^	Skin biopsies of vitiligo patients	increased secretion of mRNA of: tenascin C, gap junction beta-6 protein, thrombospondin 1 genes in the melanocytes from hair follicle bulge, increased expression of *tyrosinase* gene in the epidermal melanocytes.
Yi et al. [30]	313 nm, 90 mJ/cm^2^	50 SKH1 hairless mice	enhanced IL-6, IL-1β, and TNF-α serum levels, in skin: ✓ decreased T-SOD, and CAT levels, ✓ increased levels of MDA and collagen type 3, ✓ decreased levels of: collagen type 1 hydroxyproline, hyaluronic acid, and total protein, ✓ swelled and thickened epidermis and dermis of the skin, ✓ overstained dermis elastin ✓ increased MMP-2 and MMP-9 expression, ✓ decreased expression of TIMP-1, TIMP-2, Cu/Zn-SOD, Mn-SOD, CAT, and GSH-Px.
Gruber et al. [31]	302 nm, 300 mJ/cm^2^	MatTek^®^ Human Epidermal Skin Equivalent	increased production of IL-1α, IL-6, IL-8 and prostaglandin E_2_.
Penna et al. [32]	302 nm, 500 and 1000 mJ/cm^2^	Human Foreskin Fibroblasts	decreased cell viability, increased ROS production, cytokines level: IL1-α, IL1-β, and TNF-α,
Chen et al. [34]	290–315 nm, 1.5–50 mJ/cm^2^	Human epidermal keratinocytes (HEK) Human skin tissues	decreased mRNA of autophagy-related genes: ULK1, ATG3, and ATG7, attenuated autophagy response to MTOR signaling, ER stress, inositol pathway autophagy inducers.
Kwon et al. [36]	310 nm, 50 mJ/cm^2^ combined with 340 nm, 5 mJ/cm^2^	NC/Nga mice with induced atopic dermatitis	soothed atopic dermatitis -related lesions (edema, erythema, dryness, itching) and episodes of scratching, decreased levels of IgE, IL-4, MCP-1, IL-1β, and IL-6 in the serum.

TNFα—tumor necrosis factor α; PASI—soriasis area and severity index; T-SOD—total superoxide dismutase; CAT—catalase; MDA— malondialdehyde, IL—interleukine; MMP—matrix metalloproteinase; TIMP—tissue inhibitor of metalloproteinase; Cu/Zn-SOD, Mn-SOD—types of superoxide dismutase isozymes; GSH-Px—glutathione peroxidase; ROS—reactive oxygen species; ULK1—Unc-51 like autophagy activating kinase; ATG—autophagy-related protein; mTOR—mammalian target of rapamycin; ER—endoplasmic reticulum; IgE—immunoglobulin E; MCP-1—monocyte chemoattractant protein-1.

**Table 2:** Blue light (450–495 nm) effects on skin cells.

Author	Parameters	Material	Main Findings
Rascalou et al. [37]	450, 525 and 625 nm combined, 99 J/cm^2^	normal human fibroblasts	no cell mortality, changes in cell morphology (rounding and shrinking) cells recovered after 5 days from exposure, decreased proliferation rate, decreased synthesis of procollagen I, disorganization of the F-actin cytoskeleton, decreased ATP production.
Nakashima et al. [38]	460 nm, 0.133 J/cm^2^	hairless mice expressing roGFP1 Human normal epidermal keratinocyte (HaCaT) cells expressing roGFP1 human skin in vivo	reduced skin autofluorescence, decreased flavin autofluorescence, increased oxidative stress.
Lee et al. [39]	410 nm, 10 J/cm^2^	keloid fibroblasts isolated from keloid-revision surgery	no significant change in keloid fibroblasts viability, decreased expression and protein level of collagen I.
Mignon et al. [40]	450 nm, 0–250 J/cm^2^	primary human reticular and papillary dermal fibroblasts	decreased proliferation of papillary dermal fibroblasts, increased cytotoxic for >30 J/cm^2^ dose, increased dose-dependent ROS production, higher reticular DFs sensitivity to exposure when compared with papillary DFs, decreased production of procollagen I in reticular fibroblasts.
Zhang et al. [41]	415 nm, 432 J/cm^2^	*C. albicans* infected 16 adult mice: 8 untreated control, 8 study group	decreased the fungal burden in infected mouse burns.
Zhang et al. [42]	415 nm, 0, 28.0, 56.2, 84.2, 112.3, 140.4, and 168.5 J/cm^2^– for keratinocytes 415 nm, 55.8 J/cm^2^, 70.2 J/cm^2^, 195 J/cm^2^– for mice	Human normal epidermal keratinocyte (HaCaT), *A. baumannii* infected adult mice	decreased the bacterial burden in infected mouse burns, slightly decreased keratinocyte viability under 70 J/cm^2^.
Wang et al. [43]	460 nm, 240 J/cm^2^	*C. albicans* biofilm model, Human normal epidermal keratinocyte (HaCaT), Human normal foreskin fibroblast (Hs27), *C. albicans* infected mice	no changes in the morphology and cell viability, dose-dependent C. albicans eradication (60–240 J/cm^2^), suppressed *C. albicans* skin infection in vivo.
Makdoumi et al. [44]	450 nm, 15 J/cm^2^, 30 J/cm^2^, 56 J/cm^2^, 84 J/cm^2^	Human normal epidermal keratinocyte (HaCaT), MRSA HaCaT in vitro liquid layer model.	no effect on HaCaT cells, MRSA elimination without keratinocyte inactivation.
Mamalis et al. [45]	415 nm, 0,5,10,15, 30, 80 J/cm^2^	Primary human skin fibroblasts	decreased proliferation rate no effect on cell viability decreased migration rate increased generation of intracellular ROS
Teuschl et al. [46]	470 nm, 30 J/cm^2^	NIH/3T3 fibroblasts, BICR10 keratinocytes	decreased rate of keratinocyte proliferation no changes in fibroblast proliferation increased percentage of apoptotic and necrotic cells (fibroblasts and keratinocytes)
de Alencar Fernandes Neto et al. [47]	470 nm, 12.5 J/cm^2^	Wistar rats: control (n = 20) and blue LED (n = 20)	increased feed consumption increased angiogenic index 7 days from exposition accelerated re-epithelialization
AlGhamdi et al. [48,49]	457 nm, 0–5 J/cm^2^	Human normal, foreskin melanocytes	increased (dose-dependent manner) viability (from 0.5 to 2 J/cm^2^) increased proliferation rate (from 0.5 to 2 J/cm^2^) increased migration rate of cells higher number of stage I melanosomes
Castellano-Pellicena et al. [50]	453 nm, 2 J/cm^2^	ex vivo human skin wound healing model primary human skin keratinocytes, primary human skin dermal fibroblasts	stimulated wound healing, increased metabolic activity of keratinocytes, reduced DNA synthesis, stimulated differentiation of keratinocytes, decreased migration of keratinocytes
Marra et al. [51]	415 nm, 20 J/cm^2^	30 normal nude mouse skin	increased Stat3 crosslinking increased keratinocyte damage localized to the epidermis shrunk epidermis
Campiche et al. [52]	450 nm, 4 x 60 J/cm2	33 human female (skin phototypes III and IV)	skin hyperpigmentation increased photoaging increased melanin content increased hemoglobin concentration increased oxygen saturation

ATP—adenosine triphosphate; roGFP1—green fluorescent protein; ROS—reactive oxygen species; DFs—dermal fibroblasts; MRSA—methicillin-resistant *Staphylococcus aureus*.

**Table 3:** Green light (495–570 nm) effect on skin cells.

Author	Parameters	Material	Main Findings
Osiecka et al. [53]	540 nm, 62.5 J/cm^2^	11 patients with chronic lichen sclerosus	improved local skin status reduced pruritus symptoms
Fritsch et al. [54]	543–548 nm, 30 J/cm2	six patients with extended solar keratoses	development of erythema reported sensitivity to heat only green light ALA-PDT was superior to red light ALA-PDT
Li-qiang et al. [55]	532 nm, 96–115 J/cm^2^	82 patients with port wine stains (PWS)	post-treatment edema observed at the treated areas pain noted during treatment 24 of the 82 cases of PWS were cured, 34 cases showed good efficacy, 16 cases showed alleviation, 8 cases showed no efficacy
Zhang et al. [56]	532 nm, 9.6–15 J/cm^2^	16 patients with port wine stains (PWS)	after one treatment with HMME-PDT, two of the 16 cases of PWS were cured eight cases showed a good efficacy four cases showed alleviation two cases showed no efficacy burning sensation and pain observed in 7 cases post treatment edema observed in 15 cases
JalalKamali et al. [57]	532 nm, 1.2 J/cm^2^	basal skin carcinoma cells (BCC)	decreased cell viability
Fushimi et al. [58]	518 nm, 0.2 J/cm^2^	human normal epidermal keratinocyte (HaCaT), primary human skin dermal fibroblasts	In fibroblasts: increased mRNA expression of HGF, KGF, leptin, IL-8 and VEGF-A increased protein levels of HGF, KGF, IL-8, VEGF-A) In keratinocytes: stimulated migration of HaCat over 24 h increased mRNA expression of HB-EGF and VEGF-A increased protein levels of HB-EGF and VEGF-A
Vinck et al. [59]	570 nm, 0.1 J/cm^2^	fibroblasts from chicken embryos	increased cell proliferation
Osiecka et al. [61]	540 nm, 62.5 J/cm^2^	20 patients with actinic keratosis	complete remission in all treated areas was observed after 3 month no recurrence in areas treated with green light after 6 months 4 new AKs were observed after 9 months no erythema was observed and the slight feeling of skin tension subsided in 24 h no hyperpigmentation in green light fields

ALA-PDT—5-aminolaevulinic acid-based photodynamic therapy; PWS—port wine stains; HMME-PDT—hematoporphyrin monomethyl ether based photodynamic therapy; HGF—hepatocyte growth factor; KGF—keratinocyte growth factor; IL—interleukine; VEGF-A—vascular endothelial growth factor A; HB-EGF—heparin-binding epidermal growth factor-like growth factor.

**Table 4:** Red light (620–740 nm) effects on skin cells.

Author	Parameters	Material	Main Findings
Souza-Barros et al. [63]	635 nm, 10.75–57.6 mJ/cm^2^	40 patients	reflectance for light skin was 11.8% and for dark skin 7.9%, increased dose of laser enhanced reflectance, transmittance was decreased in dark skin compare to light – up to 4 mm thickness of the skin, the temperature was increased in light skin (0.43°C).
Gonçalves Basso et al. [74]	780 nm, 0.5, 1.5 and 3 J/cm^2^	human keratinocytes (HaCaT cell line)	increased cell migration (laser irradiation 1.5 and 3 J/cm^2^) and collagen synthesis (3 J/cm^2^), did not affect cell viability or proliferation rate.
Li et al. [64]	635 nm, 10, 20, 30 J/cm^2^	mouse fibroblasts (L929 cell line)	there were no changes in *Staphylococcus epidermis* adherence and biofilm formation, there were no changes in cytotoxicity and cell morphology.
Sperandino et al. [60]	cells: 660nm, 3, 6 or 12 J/cm^2^; animals: 660 nm, 117.85 J/cm^2^	human keratinocytes (HaCaT cell line) 40 Wistar rats	cells: increased proliferation and expression of Cyclin D1, animals: increased expression of CK10, CK14 and p63, faster maturation of the migrating keratinocytes.
Evans et al. [66]	648 nm, 1.5 J/cm^2^	human keratinocytes (CCD 1102 KERTr cell line)	increased cell proliferation and decreased in intracellular calcium while treatment with 200 µM H_2_O_2_ decreased ATP viability, intracellular calcium, and cell proliferation rate in apoptotic cells
Niu et al. [67]	combined 405 nm (1.604 J/cm^2^) and 630 nm (3.409 J/cm^2^) /660 nm, (6.538 J/cm^2^)	human keratinocytes (HaCaT cell line) treated with curcumin	decreased cell viability and cell proliferation, preserved membrane integrity, induced apoptosis by caspase activation.
Leong et al. [68]	380 to 660 nm, 1 J/cm^2^	co culture model: human keratinocytes (N/TERT-1 cell line) with human monocytic cells (THP-1 cell line)	induced release of IL-4 there were no changes in the expression of keratinocyte differentiation markers and signs of photo-oxidative damage
Hyun-Soo et al. [69]	620–690 nm, 60 J/cm^2^	normal human dermal fibroblasts (NHDF cell line)	176 genes upregulated, 57 genes downregulated, genes involved in biological response to red light: Hsp70, HSPA1A, HSPA5, PTGS2, IL-6, LIF, HMOX, ATF3, GADD45A, GADD45B, increased expression of several important genes associated with oxidative stress, wound healing, and DNA repair processes.
Song et al. [70]	628nm, 0, 0.44, 0.88, 2.00, 4.40, and 8.68 J/cm^2^	normal human fibroblasts of the newborn foreskin (HS27 cell line)	increased cell proliferation. genes expression: upregulated 68, downregulated 43. increased expression of genes associated with proliferation, migration, cell metabolism, antioxidation, DNA repair, ion and membrane channels, remodeled DNA synthesis, enhanced cell proliferation rate by suppression of apoptosis related genes.
Ayuk et al. [72]	660 nm, 5 J/cm^2^	isolated human skin fibroblast in in vitro model of diabetic wound	there were no morphological changes, increased viability, proliferation, migration, and collagen content
George et al. [71]	636 nm, 5, 10, 15, 20, 25 J/cm^2^	fibroblasts isolated from the skin of donor undergoing abdominoplasty	decreased production of ROS and increased in oxidative stress, percent of viable cells was the lowest at 15 J/cm^2^ and 20 J/cm^2^ 25 J/cm^2^ of irradiation had higher viability production of ATP was optimal until 15 J/cm^2^, but hence dropped
Fortuna et al. [73]	670 nm, 4 J/cm^2^	40 rats with scalpel-made wound	increased collagen expression, VEGF positive cells on a number of blood vessels (14-21 days of wound healing), a positive correlation of VEGF and collagen positive cells (14-28 days of wound healing).

CK—cytokeratin; p63—tumor protein 63; IL—interleukine; HSP—heat shock protein; PTGS2—prostaglandin-endoperoxide synthase 2; LIF—leukemia inhibitory factor; HMOX—heme oxygenase, ATF3—activating transcription factor 3; GADD45—growth arrest and DNA-damage-inducible protein; ROS—reactive oxygen species; VEGF—vascular endothelial growth factor.

**Table 5:** IR light (780 nm–1mm) effects on skin cells.

Author	Parameters	Material	Main Findings
George et al. [71]	825 nm, 5, 10, 15, 20, 25 J/cm^2^	Fibroblasts isolated from the skin of donor undergoing abdominoplasty	increased levels of ROS in all range of laser power, except 10 J/cm^2^, production of ATP higher in cells irradiated with 825 nm laser than the 636 nm laser, laser power above 15 J/cm^2^ damaged the functions of the mitochondria, ability to generated two types of oxide radicals.
Solmaz et al. [78]	809 nm, 1 and 3 J/cm^2^	Mouse fibroblasts (L929 cell line)	not affected cell viability, not affected wound healing.
Engel et al. [79]	808 nm, 11.3, 13.2 15.1, 17 J/cm^2^	Human oral fibroblasts, human normal oral keratinocytes-spontaneously immortalized	keratinocytes exhibited higher sensitivity to laser treatment (14.2 J/cm^2^) comparing to fibroblasts (15.1 J/cm^2^), increased production of ROS in keratinocytes than in fibroblasts, catalase activity induced by melatonin improved keratinocytes’ survival to phototoxic doses of the laser.
Schmitt et al. [80]	2940 nm, 60 J/cm^2^	3D standardized organotypic model of human skin (keratinocytes and fibroblasts isolated from patients)	increased mRNA expression of MMP1, MMP2, MMP3, TIMP1, TIMP2, CXCL1, CXCL2, CXCL5, CXCL6, IL6, IL8, and IL24, decreased mRNA expression of keratin-associated protein 4, filaggrin, filaggrin 2, and loricrin, antimicrobial peptides (S100A7A, S100A9, and S100A12), CASP14, DSG2, IL18, and IL36β, complete regeneration of the epidermis 3 days after irradiation.
De Filippis et al. [81]	1064 nm, 2, 4, 6, and 8 J/cm^2^	Human normal epidermal keratinocyte (HaCaT) Human Dermal Fibroblasts (HDF)	no influence on keratinocytes and fibroblasts morphology and viability, enhanced expression of aquaporins, filaggrin, TGase, and HSP70, after 24h of radiation increased level of proinflammatory cytokines (IL-6, IL-1α, and TNF-α) in keratinocytes, decreased level of MMP-1 and increased level of procollagen, collagen type I, and elastin in fibroblasts stimulated with irradiated keratinocyte-conditioned medium.
Asghari et.al. [82]	890 nm, 0.324 J/cm^2^	20 Wistar rats	decreased number of colony-forming units 7 days after wound induction, animals treated with laser light had better oral glucose tolerance, increased biomechanical properties of the wound, accelerated wound healing and reduced bacteria numbers.
Robati and Asadi [84]	Er:YAG laser: 2940 nm, 3.12 J/cm^2^ CO_2_ laser (far-infrared): 10600 nm, 20–18 mJ/cm^2^	40 patients	reduced facial wrinkles 3 months after the final treatment (both lasers), decreased cutaneous resonance running time, there were no serious side effects, there was no significant difference between both laser treatments.
Robati et al. [85]	Er:YAG laser: 2940 nm, 3.12 J/cm^2^ Nd:YAG laser: 1064nm, 10-20 J/cm^2^	27 patients	reduced hand wrinkles 3 months after the final treatment (both lasers), decreased cutaneous resonance running time, there were no serious side effects, there was no significant difference between both laser treatments.

ROS—reactive oxygen species, MMP—matrix metalloproteinase; ITMP—inhibitor of matrix metalloproteinase; CXCL—chemokine (C-X-C motif) ligand; IL—interleukine; TGase—Transglutaminase; HSP70—70 kilodalton heat shock protein; S100—calcium binding protein; CASP14—caspase 14, DSG2—desmoglein 2.

## Abbreviations

IR	infrared
UV	ultraviolet
ROS	reactive oxygen species
HDF	human dermal fibroblast
HaCaT	human normal epidermal keratinocytes cell lines
MMP	matrix metalloproteinase
IL	interleukin
NIR	near infrared
